# Tetrabutylammonium Iodide–Promoted Thiolation of Oxindoles Using Sulfonyl Chlorides as Sulfenylation Reagents

**DOI:** 10.3390/molecules22081208

**Published:** 2017-08-01

**Authors:** Xia Zhao, Aoqi Wei, Xiaoyu Lu, Kui Lu

**Affiliations:** 1 College of Chemistry, Tianjin Key Laboratory of Structure and Performance for Functional Molecules, Key laboratory of Inorganic-organic Hybrid Functional Material Chemistry, Ministry of Education, Tianjin Normal University, Tianjin 300387, China; weiaoqi920209@163.com (A.W.); lxy595380904@126.com (X.L.); 2 College of Biotechnology, Tianjin University of Science & Technology, Tianjin 300457, China; lukui@tust.edu.cn

**Keywords:** thiolation, oxindole, sulfonyl chloride, tetrabutylammonium iodide, triphenylphosphine

## Abstract

3-Sulfanyloxindoles were synthesised by triphenylphosphine-mediated transition-metal-free thiolation of oxindoles using sulfonyl chlorides as sulfenylation reagents. The above reaction was promoted by iodide anions, which was ascribed to the in situ conversion of sulfenyl chlorides into the more reactive sulfenyl iodides. Moreover, the thiolation of 3-aryloxindoles was facilitated by bases. The use of a transition-metal-free protocol, readily available reagents, and mild reaction conditions make this protocol more practical for preparing 3-sulfanyloxindoles than traditional methods.

## 1. Introduction

**Figure 1:**
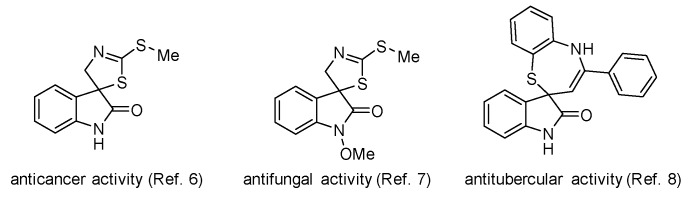
Bioactive oxindoles with thiolation at the C-3 position.

**Scheme 1:**
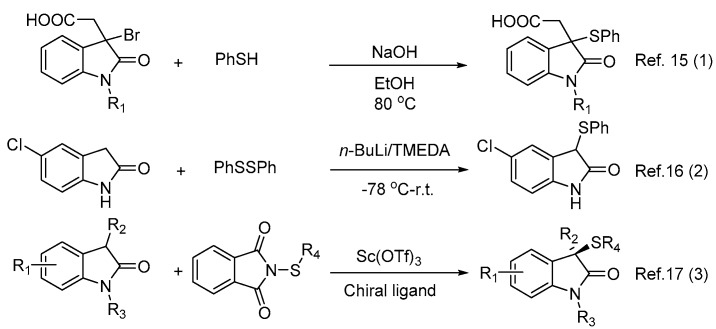
Previously reported syntheses of 3-sulfanyloxindoles.

Oxindoles and their derivatives have attracted increased attention as a frequently occurring structural motif of both natural products and bioactive compounds [1,2,3,4,5], with thiolation at the C-3 position imparting anticancer [6], antifungal [7], and antitubercular activities (Figure 1) [8]. Therefore, the synthesis of 3-sulfanyloxindoles has been widely investigated, including with methods such as cyclisation of sulfur-containing compounds [9,10,11,12,13,14], nucleophilic substitution reactions of 3-bromooxindoles (Scheme 1, Equation (1)) [15], electrophilic thiolation of oxindoles with sulfinothioyldibenzene (Scheme 1, Equation (2)) [16] and electrophilic thiolation of oxindoles with *N*-(arylthio)phthalimides (Scheme 1, Equation (3)) [17,18]. Although electrophilic thiolation is the most straightforward method, the need for strongly basic conditions and the limited availability of sulfenylation reagents limit its further application.

**Scheme 2:**
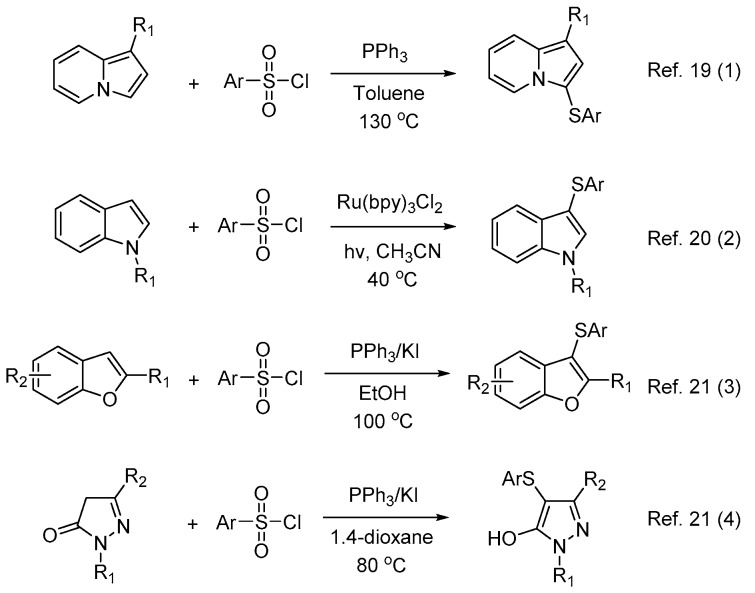
Thiolation of electron-rich aromatics using sulfonyl chlorides as sulfenylation reagents.

**Scheme 3:**
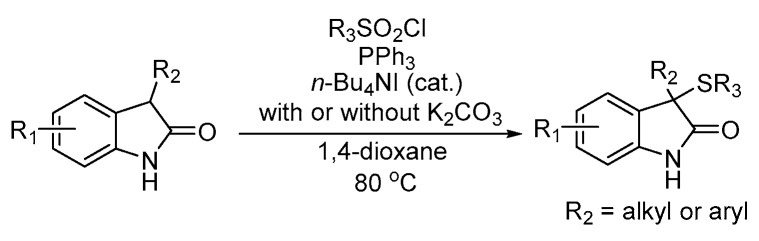
Present Results for Thiolation of oxindoles by sulfonyl chlorides in the presence of PPh_3_.

Recently, the use of sulfonyl chlorides as sulfenylation reagents has been reported by You (Scheme 2, Equation (1)) [19], Zheng (Scheme 2, Equation (2)) [20] and our group (Scheme 2, Equations (3) and (4)) [21]. As a part of our on-going development of new sulfenylation methods [21,22,23,24,25,26,27,28], we report here a novel tetrabutylammonium iodide-facilitated thiolation of oxindoles with sulfonyl chlorides as sulfenylation reagents (Scheme 3).

## 2. Results

Treatment of 3-methylindolin-2-one (**1a**) with 4-methylbenzenesulfonyl chloride (**2a**) in the presence of PPh_3_ in 1,4-dioxane at 80 °C afforded 3-methyl-3-(*p*-tolylthio)indolin-2-one (**3aa)** in 56% yield (Table 1, Entry 1). In agreement with our previous studies, this transformation was facilitated by iodide anions [21]. Therefore, a number of classical iodides were initially screened, including potassium iodide (KI), ammonium iodide (NH_4_I), and tetrabutylammonium iodide (*n*-Bu_4_NI), with the highest yield observed for *n*-Bu_4_NI (Table 1, Entries 2–4). Subsequently, other solvents, such as 1,2-dichloroethane (DCE), toluene, acetonitrile (CH_3_CN), and *N*,*N*-dimethylformamide (DMF) were tested, but none of them surpassed 1,4-dioxane (Table 1, Entries 5–8). Finally, the effects of temperature and concentration were examined, revealing that decreasing the reaction temperature to 70 °C or increasing it to 90 °C diminished the yield (Table 1, Entries 9 and 10), as was also observed for decreasing the concentration of **1a** from 0.5 M to 0.33 M (Table 1, Entry 11). When the concentration of **1a** was increased from 0.5 M to 1.0 M, the desired product was obtained in 86% yield (Table 1, Entry 12), with further concentration increases leading to diminished yields (Table 1, Entry 13). Notably, increasing the loadings of **2a** and PPh_3_ to 1.5 and 3.0 equiv., respectively, did not significantly affect the yield (Table 1, Entry 14). Thus, the optimised reaction conditions for the thiolation of **1a** were as follows: **1a** (0.5 mmol), **2a** (0.6 mmol), PPh_3_ (1.0 mmol), *n*-Bu_4_NI (0.1 mmol), and 1,4-dioxane (0.5 mL) at 80 °C.

The optimised conditions were used to investigate the substrate scope of sulfenylation. As shown in Table 2, a series of substituted 3-alkyloxindoles could be coupled with various sulfonyl chlorides to afford the corresponding oxindole thioethers in moderate to excellent yields, with 3-alkyl-(**1a**, **1c**–**1g**), 3-benzyl-(**1h** and **1i**), and 5-bromo-substituted (**1b**) oxindoles being well tolerated. In the case of aromatic sulfonyl chlorides, both electron-donating and electron-withdrawing groups, as well as diverse *ortho*-, *meta*-, and *para*-substituents (**2b**–**2e**) were tolerated. Notably, for electronic effect, aliphatic sulfonyl chlorides (**2f** and **2g**) provided the desired thiolation products in a relatively low yield compared with aromatic sulfonyl chlorides.

To further extend the substrate scope of this reaction, we explored the thiolation of 3-aryl-substituted oxindoles with sulfonyl chlorides using 3-(*p*-tolyl)indolin-2-one (**4a**) and 3-chlorobenzenesulfonyl chloride (**2h**) as model substrates in the presence of PPh_3_ under optimised reaction conditions. However, no desired product (**5ah**) was obtained (Table 3, Entry 1). Fortunately, when the reaction was carried out at 60 °C, **5ah** was obtained in 44% yield (Table 3, Entry 2). As a further optimisation, potassium carbonate was employed as a base to activate the substrate, affording a significantly improved yield, especially when the reaction was carried out at 40 °C (Table 3, Entries 5–8). Subsequently, other bases, base loadings, additives, thiolation reagents, and reductants were tested, with the optimal reaction condition identified as: **4a** (0.25 mmol), **2h** (0.3 mmol), PPh_3_ (0.5 mmol), *n*-Bu_4_NI (0.05 mmol), K_2_CO_3_ (0.125 mmol), and 1,4-dioxane (1.0 mL) at 40 °C.

**Figure 2:**
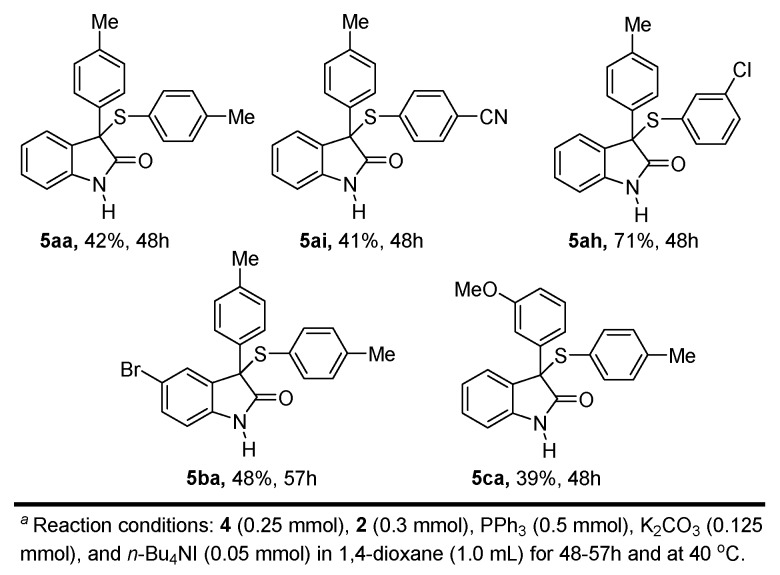
Thiolation of 3-aryloxindoles with sulfonyl chlorides in the presence of PPh_3_.^a^

With the new optimised conditions in hand, the generality of the thiolation reaction was examined using various 3-aryloxindoles and arylsulfonyl chlorides (Figure 2), with the desired sulfenylation products (**5aa**–**5ca**) obtained in moderate yields.

## 3. Discussion

**Scheme 4:**
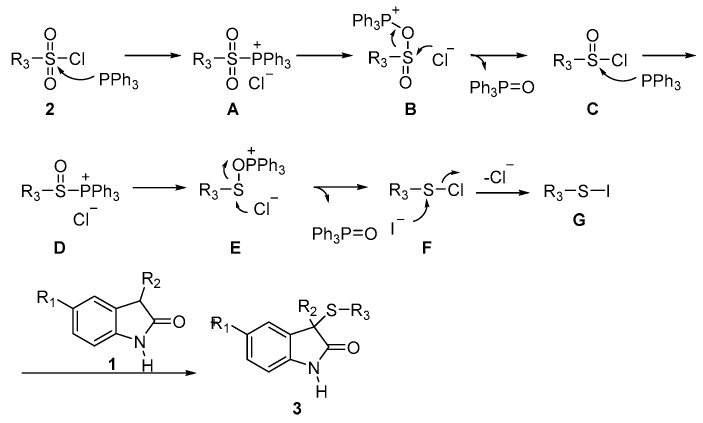
Proposed reaction mechanism.

Based on our previous work [21], a plausible reaction mechanism was proposed (Scheme 4), featuring the initial reduction of sulfonyl chloride **2** by PPh_3_ to sulfenyl chloride **F** via intermediates **A**–**E**. **F** is converted into sulfenyl iodide **G** in the presence of iodide anions. Finally, electrophilic thiolation of oxindoles **1** by **G** gives the corresponding oxindole thioethers.

## 4. Materials and Methods

### 4.1. General Methods and Material

All solvents were distilled prior to use. Unless otherwise noted, chemicals were used as received without further purification. For chromatography, 200−300 mesh silica gel was employed. ^1^H- and ^13^C-NMR spectra were recorded at 400 MHz and 100 MHz respectively. Chemical shifts are reported in ppm using tetramethylsilane as internal standard (see supplementary). HRMS was performed on an FTMS mass instrument. Melting points are reported as uncorrected.

### 4.2. Synthesis of Oxindoles

**1c–1i** were synthesized according to the literature procedures [29].

#### 4.2.1. 5-Bromo-3-methylindolin-2-one (**1b**)

3-methylindolin-2-one (441 mg, 3 mmol) in acetonitrile (5 mL) was cooled to −15°C. NBS (534 mg, 3 mmol) was added. After stirring for 1 h, the reaction was diluted with water (10 mL) and extracted with EtOAc (20 mL) for three times. The combined organic phase was washed with brine, dried over Na_2_SO_4_ and concentrated under reduced pressure to give a residue which was purified by silica gel column chromatography to afford compound **1b** (454 mg, 67%) as a white solid.

#### 4.2.2. 3-(*p*-Tolyl)indolin-2-one (**4a**)

Indoline-2,3-dione (1.47 g, 10 mmol) in THF (20 mL) was cooled to −15°C. NaH (60%/mineral oil, 600 mg, 15 mmol) was added. After stirring for 30 min, *p*-tolylmagnesium bromide (1.0 M/THF, 10 mL, 10 mmol) was added. The reaction mixture was allowed to warm to room temperature and stirred for 1h. Then the reaction was quenched with NH4Cl (aq) (30 mL) and extracted with Et_2_O (50 mL) for three times. After stirring for 1 h, the reaction was diluted with water (10 mL) and extracted with EtOAc (20 mL) three times. The combined organic phase was washed with brine, dried over Na_2_SO_4_ and concentrated under reduced pressure to give a residue, which was purified by silica gel column chromatography to afford compound **1b** (454 mg, 67%) as a yellow solid.

### 4.3. General Procedure for the Synthesis of ***3aa***, ***3ab***, ***3ac***, ***3ad***, ***3ae***, ***3af***, ***3ag***, ***3ba***, ***3ca*** and ***3da***

Oxindole (0.5 mmol), sulfonyl chloride (0.6 mmol), PPh_3_ (1.0 mmol), *n*-Bu_4_NI (0.1 mmol) and dry 1,4-dioxane (0.5 mL) were mixed in an oven dried sealed tube. The mixture was stirred at 80 °C for 12 h. Then, the solvent was evaporated under reduced pressure and the residue was purified by silica gel column chromatography (PE:EA = 5:1 or PE:EA = 3:1) to afford the pure product.

### 4.4. General Procedure for the Synthesis of ***3ea***, ***3fa***, ***3ga***, ***3gb***, ***3ha*** and ***3ia***

Oxindole (0.25 mmol), sulfonyl chloride (0.3 mmol), PPh_3_ (0.5 mmol), *n*-Bu_4_NI (0.05 mmol) and dry 1,4-dioxane (0.25 mL) were mixed in an oven dried sealed tube. The mixture was stirred at 80 °C for 6–30 h. Then, the solvent was evaporated under reduced pressure and the residue was purified by silica gel column chromatography (PE:EA = 5:1, PE:EA = 4:1 or PE:EA = 3:1) to afford the pure product.

### 4.5. General Procedure for the Synthesis of ***5aa***, ***5ai***, ***5ah***, ***5ba*** and ***5ca***

Oxindole (0.25 mmol), sulfonyl chloride (0.3 mmol), PPh_3_ (0.5 mmol), *n*-Bu_4_NI (0.05 mmol), K_2_CO_3_ (0.125 mmol) and dry 1,4-dioxane (1.0 mL) were mixed in an oven-dried sealed tube. The mixture was stirred at 40 °C for the time indicated. Then, the solvent was evaporated under reduced pressure and the residue was purified by silica gel column chromatography (PE:EA = 5:1 or PE:EA = 3:1) to afford the pure product.

3-Methyl-3-(*p*-tolylthio)indolin-2-one (**3aa**). After purification by silica gel column chromatography (PE:EA = 5:1), compound **3aa** was isolated as a white solid (116 mg, 86%); m.p. = 151–152 °C; *R*_f_ (PE:EA = 3:1) = 0.32; ^1^H-NMR (400 MHz, CDCl_3_): δ 8.39 (s, 1H), 7.35 (d, *J* = 7.4 Hz, 1H), 7.15 (td, *J* = 7.7 Hz, 1.3 Hz, 1H), 7.11 (d, *J* = 8.0 Hz, 2H), 7.07 (td, *J* = 7.5 Hz, 1.0 Hz, 1H), 6.91 (d, *J* = 7.9 Hz, 2H), 6.70 (d, *J* = 7.7 Hz, 1H), 2.24 (s, 3H), 1.69 (s, 3H); ^13^C-NMR (100 MHz, CDCl_3_): δ 179.3, 139.8, 139.6, 136.3, 132.1, 129.2, 128.6, 126.4, 124.2, 122.6, 109.7, 54.9, 21.4, 21.2; HRMS (ESI) *m*/*e* calcd. for C_16_H_15_NOS (M + H)^+^ 270.0947, found 270.0947.

3-[(4-Methoxyphenyl)thio]-3-methylindolin-2-one (**3ab**). After purification by silica gel column chromatography (PE:EA = 3:1), compound **3ab** was isolated as a pink solid (128 mg, 90%); m.p. = 153–154 °C; *R*_f_ (PE:EA = 3:1) = 0.27; ^1^H-NMR (400 MHz, CDCl_3_): δ 7.67 (s, 1H), 7.36 (d, *J* = 7.4 Hz, 1H), 7.17–7.13 (m, 3H), 7.07 (td, *J* = 7.6 Hz, 1.0 Hz, 1H), 6.67–6.62 (m, 3H), 3.72 (s, 3H), 1.69 (s, 3H); ^13^C-NMR (100 MHz, CDCl_3_): δ 179.6, 160.7, 140.0, 137.9, 132.1, 128.6, 124.1, 122.6, 120.7, 113.9, 109.8, 55.1, 55.1, 21.1; HRMS (ESI) *m*/*e* calcd. for C_16_H_15_NO2S (M + H)^+^ 286.0896, found 286.0896.

3-Methyl-3-(*m*-tolylthio)indolin-2-one (**3ac**). After purification by silica gel column chromatography (PE:EA = 5:1), compound **3ac** was isolated as a pale solid (83 mg, 62%); m.p. = 106–107 °C; *R*_f_ (PE:EA = 3:1) = 0.45; ^1^H-NMR (400 MHz, CDCl_3_): δ 7.71 (s, 1H), 7.22 (t, *J* = 7.2 Hz, 2H), 7.17–7.09 (m, 3H), 7.02 (t, *J* = 7.5 Hz, 1H), 6.94 (td, *J* = 7.6 Hz, 1.1 Hz, 1H), 6.69 (d, *J* = 7.7 Hz, 1H), 2.30 (s, 3H), 1.74 (s, 3H); ^13^C-NMR (100 MHz, CDCl_3_): δ 179.6, 143.7, 139.9, 137.5, 131.9, 130.2, 129.6, 129.4, 128.7, 125.7, 124.1, 122.4, 109.9, 55.0, 21.7, 21.0; HRMS (ESI) *m*/*e* calcd. for C_16_H_15_NOS (M + H)^+^ 270.0947, found 270.0946.

3-((3,5-Dichlorophenyl)thio)-3-methylindolin-2-one (**3ad**). After purification by silica gel column chromatography (PE:EA = 5:1), compound **3ad** was isolated as a white solid (110 mg, 68%); m.p. = 176–177 °C; *R*_f_ (PE:EA = 5:1) = 0.30; ^1^H-NMR (400 MHz, *d*_6_-DMSO): δ 10.50 (s, 1H), 7.56 (s, 1H), 7.38 (d, *J* = 7.4 Hz, 1H), 7.18 (t, *J* = 7.6 Hz, 1H), 7.10 (d, *J* = 1.8 Hz, 2H), 7.03 (t, *J* = 7.5 Hz, 1H), 6.71 (d, *J* = 7.7 Hz, 1H), 1.58 (s, 3H); ^13^C-NMR (100 MHz, *d*_6_-DMSO): δ 177.0, 141.1, 133.9, 133.6, 133.1, 130.7, 129.3, 129.0, 124.1, 122.1, 109.7, 54.8, 21.3; HRMS (ESI) *m*/*e* calcd. for C_15_H_11_Cl_2_NOS (M + H)^+^ 324.0011, found 324.0010.

3-((4-Bromophenyl)thio)-3-methylindolin-2-one (**3ae**). After purification by silica gel column chromatography (PE:EA = 5:1), compound **3ae** was isolated as a white solid (136 mg, 82%); m.p. = 135–137 °C; *R*_f_ (PE:EA = 3:1) = 0.40; ^1^H-NMR (400 MHz, CDCl_3_): δ 7.65 (s, 1H), 7.38 (d, *J* = 7.4 Hz, 1H), 7.25–7.23 (m, 2H), 7.17 (td, *J* = 7.7 Hz, 1.3 Hz, 1H), 7.11–7.07 (m, 3H), 6.68 (d, *J* = 7.7 Hz, 1H), 1.70 (s, 3H); ^13^C-NMR (100 MHz, CDCl_3_): δ 179.0, 139.8, 137.7, 131.7, 131.6, 129.0, 128.9, 124.4, 124.2, 122.8, 110.0, 55.1, 21.5; HRMS (ESI) *m*/*e* calcd. for C_15_H_12_BrNOS (M + H)^+^ 333.9895, found 333.9895.

3-(Cyclopropylthio)-3-methylindolin-2-one (**3af**). After purification by silica gel column chromatography (PE:EA = 5:1), compound **3af** was isolated as a white solid (48 mg, 44%); m.p. = 122–124 °C; *R*_f_ (PE:EA = 3:1) = 0.30; ^1^H-NMR (400 MHz, CDCl_3_): δ 9.57 (s, 1H), 7.34 (d, *J* = 7.4 Hz, 1H), 7.24 (td, *J* = 7.6 Hz, 0.8 Hz, 1H), 7.08 (t, *J* = 7.5 Hz, 1H), 6.98 (d, *J* = 7.7 Hz, 1H), 1.67 (s, 3H), 1.62–1.56 (s, 1H), 0.72–0.66 (s, 1H), 0.63–0.51 (m, 2H), 0.35–0.28 (m, 1H); ^13^C-NMR (100 MHz, CDCl_3_): δ 181.3, 140.1, 132.5, 128.6, 123.9, 122.8, 110.1, 52.5, 21.9, 10.1, 7.53, 5.65; HRMS (ESI) *m*/*e* calcd. for C_12_H_13_NOS (M + H)^+^ 220.0790, found 220.0789.

3-(Butylthio)-3-methylindolin-2-one (**3ag**). After purification by silica gel column chromatography (PE:EA = 5:1), compound **3ag** was isolated as a yellow liquid (66 mg, 56%); *R*_f_ (PE:EA = 3:1) = 0.42; ^1^H-NMR (400 MHz, CDCl_3_): δ 8.67 (s, 1H), 7.33 (d, *J* = 7.4 Hz, 1H), 7.23 (td, *J* = 7.7 Hz, 1.2 Hz, 1H), 7.09 (td, *J* = 7.6 Hz, 0.7 Hz, 1H), 6.93 (d, *J* = 7.7 Hz, 1H), 2.44 (dt, *J* = 11.6 Hz, 7.3 Hz, 1H), 2.28 (dt, *J* = 11.6 Hz, 7.4 Hz, 1H), 1.67 (s, 3H), 1.41–1.36 (m, 2H), 1.32–1.25 (m, 2H), 0.79 (t, *J* = 7.3 Hz, 3H); ^13^C-NMR (100 MHz, CDCl_3_): δ 180.1, 139.7, 132.3, 128.7, 124.0, 123.0, 109.8, 50.8, 30.8, 28.8, 22.4, 22.0, 13.5; HRMS (ESI) *m*/*e* calcd. for C_13_H_17_NOS (M + H)^+^ 236.1103, found 236.1103.

5-Bromo-3-methyl-3-(p-tolylthio)indolin-2-one (**3ba**). After purification by silica gel column chromatography (PE:EA = 3:1), compound **3ba** was isolated as a pale solid (156 mg, 90%); m.p. = 167–168 °C; *R*_f_ (PE:EA = 3:1) = 0.33; ^1^H-NMR (400 MHz, CDCl_3_): δ 8.58 (s,1H), 7.43 (d, *J* = 1.9 Hz, 1H), 7.28 (dd, *J* = 8.2 Hz, *J* = 2.0 Hz, 1H), 7.12 (d, *J* = 8.1 Hz, 2H), 6.95 (d, *J* = 7.9 Hz, 2H), 6.60 (d, *J* = 8.3 Hz, 1H), 2.26 (s, 3H), 1.68 (s, 3H); ^13^C-NMR (100 MHz, CDCl_3_): δ 179.6, 139.9, 138.9, 136.2, 134.1, 131.4, 129.3, 127.2, 125.8, 115.1, 111.5, 55.0, 21.2, 21.2; HRMS (ESI) *m*/*e* calcd. for C_16_H_14_BrNOS (M + H)^+^ 348.0052, found 348.0052.

3-Ethyl-3-(p-tolylthio)indolin-2-one (**3ca**). After purification by silica gel column chromatography (PE:EA = 5:1), compound **3ca** was isolated as a white solid (106 mg, 79%); m.p. = 178–179 °C; *R*_f_ (PE:EA = 3:1) = 0.37; ^1^H-NMR (400 MHz, CDCl_3_): δ 7.91 (s, 1H), 7.32 (d, *J* = 7.4 Hz, 1H), 7.15 (td, *J* = 7.6 Hz, 1.3 Hz, 1H), 7.12 (d, *J* = 8.1 Hz, 2H), 7.07 (td, *J* = 7.5 Hz, 1.0 Hz, 1H), 6.91 (d, *J* = 7.9 Hz, 2H), 6.67 (d, *J* = 7.7 Hz, 1H), 2.24 (s, 3H), 2.23–2.09 (m, 2H), 0.76 (t, *J* = 7.4 Hz, 3H); ^13^C-NMR (100 MHz, CDCl_3_): δ 179.0, 140.8, 139.5, 136.4, 123.0, 129.1, 128.5, 126.0, 124.4, 122.5, 109.8, 60.0, 28.5, 21.2, 9.23; HRMS (ESI) *m*/*e* calcd. for C_17_H_17_NOS (M + H)^+^ 284.1103, found 284.1105.

3-Propyl-3-(*p*-tolylthio)indolin-2-one (**3da**). After purification by silica gel column chromatography (PE:EA = 5:1), compound **3da** was isolated as a pale solid (74 mg, 81%); m.p. = 152–153 °C; *R*_f_ (PE:EA = 3:1) = 0.40; ^1^H-NMR (400 MHz, CDCl_3_): δ 8.67 (s, 1H), 7.32 (d, *J* = 7.3 Hz, 1H), 7.15 (td, *J* = 7.6 Hz, 1.3 Hz, 1H), 7.10 (d, *J* = 8.1 Hz, 2H), 7.06 (td, *J* = 7.5 Hz, 0.8 Hz, 1H), 6.89 (d, *J* = 7.9 Hz, 2H), 6.70 (d, *J* = 7.6 Hz, 1H), 2.22 (s, 3H), 2.17–2.01 (m, 2H), 1.20–1.05 (m, 2H), 0.84 (t, *J* = 7.3 Hz, 3H); ^13^C-NMR (100 MHz, CDCl_3_): δ 178.9, 140.6, 139.5, 136.4, 130.4, 129.1, 128.5, 126.0, 124.5, 122.5, 109.7, 59.4, 37.4, 21.2, 18.3, 14.0; HRMS (ESI) *m*/*e* calcd. for C_18_H_19_NOS (M + H)^+^ 298.1260, found 298.1267.

3-Isopropyl-3-(*p*-tolylthio)indolin-2-one (**3ea**). After purification by silica gel column chromatography (PE:EA = 5:1), compound **3ea** was isolated as a white solid (47 mg, 63%); m.p. = 162–163 °C; *R*_f_ (PE:EA = 5:1) = 0.32; ^1^H-NMR (400 MHz, CDCl_3_): δ 8.45 (s, 1H), 7.44 (d, *J* = 7.4 Hz, 1H), 7.14 (td, *J* = 7.6 Hz, 1.2 Hz, 1H), 7.07–7.03 (m, 3H), 6.85 (d, *J* = 7.8 Hz, 2H), 6.66 (d, *J* = 7.6 Hz, 1H), 2.47 (h, *J* = 6.8 Hz, 1H), 2.20 (s, 3H), 1.28 (d, *J* = 7.0 Hz, 3H), 0.87 (d, *J* = 6.8 Hz, 3H); ^13^C-NMR (100 MHz, CDCl_3_): δ 179.2, 140.8, 139.3, 136.2, 129.2, 129.1, 128.4, 126.0, 125.5, 122.2, 109.7, 64.1, 33.8, 21.1, 18.0, 17.7; HRMS (ESI) *m*/*e* calcd. for C_18_H_19_NOS (M + H)^+^ 298.1260, found 298.1259.

3-Isopentyl-3-(*p*-tolylthio)indolin-2-one (**3fa**). After purification by silica gel column chromatography (PE:EA = 5:1), compound **3fa** was isolated as a white solid (64 mg, 78%); m.p. = 159–160 °C; *R*_f_ (PE:EA = 5:1) = 0.30; ^1^H-NMR (400 MHz, CDCl_3_): δ 7.91 (s, 1H), 7.32 (d, *J* = 7.3 Hz, 1H), 7.14 (td, *J* = 7.6 Hz, 1.2 Hz, 1H), 7.10–7.05 (m, 3H), 6.90 (d, *J* = 7.9 Hz, 2H), 6.65 (d, *J* = 7.6 Hz, 1H), 2.23 (s, 3H), 2.20–2.04 (m, 2H), 1.51–1.44 (m, 1H), 1.09–0.86 (m, 2H), 0.81 (d, *J* = 6.6 Hz, 6H), 0.80 (s, 3H); ^13^C-NMR (100 MHz, CDCl_3_): δ 178.9, 140.6, 139.5, 136.4, 130.4, 129.1, 128.4, 126.0, 124.4, 122.5, 109.7, 59.4, 33.5, 33.2, 28.1, 22.4, 22.2, 21.2; HRMS (ESI) *m*/*e* calcd. for C_20_H_23_NOS (M + H)^+^ 326.1573, found 326.1570.

3-Cyclohexyl-3-(*p*-tolylthio)indolin-2-one (**3ga**). After purification by silica gel column chromatography (PE:EA = 4:1), compound **3ga** was isolated as a white solid (56 mg, 67%); m.p. = 216–217 °C; *R*_f_ (PE:EA = 3:1) = 0.47; ^1^H-NMR (400 MHz, *d*_6_-DMSO): δ 10.2 (s, 1H), 7.35 (d, *J* = 7.4 Hz, 1H), 7.11 (t, *J* = 7.3 Hz, 1H), 7.00–6.93 (m, 5H), 6.58 (d, *J* = 7.6 Hz, 1H), 2.19 (s, 3H), 2.04 (d, *J* = 12 Hz, 1H), 1.96 (d, *J* = 11.8 Hz, 1H), 1.76 (d, *J* = 12.2 Hz, 1H), 1.59–1.53 (m, 3H), 1.35–1.10 (m, 3H), 1.04–0.97 (m, 1H), 0.86–0.76 (m, 1H); ^13^C-NMR (100 MHz, *d*_6_-DMSO): δ 176.5, 141.8, 138.7, 135.7, 129.2, 129.0, 128.4, 126.0, 125.1, 121.4, 109.1, 63.2, 43.3, 27.6, 27.2, 25.9, 25.7, 25.7, 20.6; HRMS (ESI) *m*/*e* calcd. for C_21_H_23_NOS (M + H)^+^ 338.1573, found 338.1572.

3-Cyclohexyl-3-((4-methoxyphenyl)thio)indolin-2-one (**3gb**). After purification by silica gel column chromatography (PE:EA = 5:1), compound **3gb** was isolated as a white solid (52 mg, 65%); m.p. = 198–199 °C; *R*_f_ (PE:EA = 3:1) = 0.42; ^1^H-NMR (400 MHz, CDCl_3_): δ 7.76 (s, 1H), 7.45 (d, *J* = 7.3 Hz, 1H), 7.13 (td, *J* = 7.6 Hz, 1.1 Hz, 1H), 7.08–7.04 (m, 3H), 6.60 (d, *J* = 7.7 Hz, 1H), 6.57 (d, *J* = 8.8 Hz, 2H), 3.68 (s, 3H), 2.21 (m, 2H), 1.83 (d, *J* = 12.6 Hz, 1H), 1.64 (d, *J* = 10.6 Hz, 2H), 1.42–1.22 (m, 4H), 1.13–0.88 (m, 2H); ^13^C-NMR (100 MHz, CDCl_3_): δ 179.0, 160.4, 140.7, 137.9, 129.9, 128.3, 125.6, 122.2, 120.0, 113.7, 109.6, 64.3, 55.0, 43.7, 28.3, 27.8, 26.5, 26.2, 26.1; HRMS (ESI) *m*/*e* calcd. for C_21_H_23_NO_2_S (M + H)^+^ 354.1522, found 354.1522.

4-((2-Oxo-3-(*p*-tolylthio)indolin-3-yl)methyl)benzonitrile (**3ha**). After purification by silica gel column chromatography (PE:EA = 3:1), compound **3ha** was isolated as a white solid (56 mg, 60%); m.p. = 238–239 °C; *R*_f_ (PE:EA = 3:1) = 0.26; ^1^H-NMR (400 MHz, *d*_6_-DMSO): δ 10.17 (s, 1H), 7.56 (d, *J* = 8.3 Hz, 2H), 7.45 (d, *J* = 7.0 Hz, 1H), 7.11 (d, *J* = 8.2 Hz, 4H), 7.07–7.03 (m, 3H), 7.00 (td, *J* = 7.5 Hz, 0.8 Hz, 1H), 6.44 (d, *J* = 7.5 Hz, 1H), 3.52 (d, *J* = 12.9 Hz, 1H), 3.35 (d, *J* = 12.9 Hz, 1H), 2.24 (s, 3H); ^13^C-NMR (100 MHz, *d*_6_-DMSO): δ 175.8, 141.3, 141.2, 139.4, 136.1, 131.6, 130.9, 129.2, 128.9, 128.1, 125.6, 125.0, 121.5, 118.5, 109.6, 109.4, 59.2, 39.9, 20.7; HRMS (ESI) *m*/*e* calcd. for C_23_H_18_N_2_OS (M + H)^+^ 371.1212, found 371.1213.

3-(4-Chlorobenzyl)-3-(*p*-tolylthio)indolin-2-one (**3ia**). After purification by silica gel column chromatography (PE:EA = 5:1), compound **3ia** was isolated as a white solid (75 mg, 79%); m.p. = 217–218 °C; *R*_f_ (PE:EA = 3:1) = 0.50; ^1^H-NMR (400 MHz, *d*_6_-DMSO): δ 10.1 (s, 1H), 7.42 (d, *J* = 7.1 Hz, 1H), 7.13 (d, *J* = 8.4 Hz, 2H), 7.10 (d, *J* = 8.1 Hz, 2H), 7.07–7.02 (m, 3H), 6.97 (t, *J* = 6.7 Hz, 1H), 6.92 (d, *J* = 8.4 Hz, 2H), 6.45 (d, *J* = 7.6 Hz, 1H), 3.40 (d, *J* = 13.0 Hz, 1H), 3.26 (d, *J* = 13.0 Hz, 1H), 2.24 (s, 3H); ^13^C-NMR (100 MHz, *d*_6_-DMSO): δ 176.1, 141.5, 139.4, 136.1, 134.4, 131.7, 131.5, 129.0, 128.8, 128.5, 127.8, 125.8, 125.05, 121.5, 109.3, 59.4, 39.5, 20.8; HRMS (ESI) *m*/*e* calcd. for C_22_H_18_ClNOS (M + H)^+^ 380.0870, found 380.0870.

3-(*p*-Tolyl)-3-(*p*-tolylthio)indolin-2-one (**5aa**). After purification by silica gel column chromatography (PE:EA = 5:1), compound **5aa** was isolated as a white solid (37 mg, 42%); m.p. = 196–197 °C; *R*_f_ (PE:EA = 3:1) = 0.41; ^1^H-NMR (400 MHz, CDCl_3_): δ 7.65 (s, 1H), 7.59 (d, *J* = 8.2 Hz, 2H), 7.40 (d, *J* = 7.4 Hz, 1H), 7.17 (d, *J* = 8.5 Hz, 2H), 7.16–7.10 (m, 2H), 7.08 (d, *J* = 8.0 Hz, 2H), 6.87 (d, *J* = 7.9 Hz, 2H), 6.64 (d, *J* = 7.6 Hz, 1H), 2.34 (s, 3H), 2.22 (s, 3H); ^13^C-NMR (100 MHz, CDCl_3_): δ 177.8, 140.3, 139.6, 138.0, 136.2, 133.2, 130.8, 129.3, 129.1, 128.6, 127.9, 126.5, 126.3, 122.5, 110.1, 62.8, 21.2, 21.0; HRMS (ESI) *m*/*e* calcd. for C_22_H_19_NOS (M + H)^+^ 346.1260, found 346.1260.

4-((2-Oxo-3-(*p*-tolyl)indolin-3-yl)thio)benzonitrile (**5ai**). After purification by silica gel column chromatography (PE:EA = 5:1), compound **5ai** was isolated as a pale solid (37 mg, 41%); m.p. = 178–181 °C; *R*_f_ (PE:EA = 3:1) = 0.36; ^1^H-NMR (400 MHz, CDCl_3_): δ 7.64 (s, 1H), 7.56 (d, *J* = 8.3 Hz, 2H), 7.42 (d, *J* = 7.5 Hz, 1H), 7.35 (d, *J* = 8.4 Hz, 2H), 7.30 (d, *J* = 8.4 Hz, 2H), 7.24–7.19 (m, 3H), 7.12 (t, *J* = 7.6 Hz, 1H), 6.71 (d, *J* = 7.7 Hz, 1H), 2.35 (s, 3H); ^13^C-NMR (100 MHz, CDCl_3_): δ 177.0, 139.9, 138.7, 137.1, 135.5, 132.3, 131.7, 129.8, 129.6, 129.35, 127.72, 126.3, 123.0, 118.2, 112.5, 110.3, 62.6, 21.1; HRMS (ESI) *m*/*e* calcd. for C_22_H_16_N_2_OS (M + H)^+^ 357.1056, found 357.1058.

3-((3-Chlorophenyl)thio)-3-(*p*-tolyl)indolin-2-one (**5ah**). After purification by silica gel column chromatography (PE:EA = 5:1), compound **5ah** was isolated as a white solid (65 mg, 71%); m.p. = 192–193 °C; *R*_f_ (PE:EA = 3:1) = 0.40; ^1^H-NMR (400 MHz, CDCl_3_): δ 7.68 (s, 1H), 7.58 (d, *J* = 8.2 Hz, 2H), 7.43 (d, *J* = 7.3 Hz, 1H), 7.22–7.12 (m, 5H), 7.19 (d, *J* = 7.8 Hz, 2H), 7.00 (t, *J* = 7.9 Hz, 1H), 6.68 (d, *J* = 7.6 Hz, 1H), 2.35 (s, 3H); ^13^C-NMR (100 MHz, CDCl_3_): δ 177.3, 140.1, 138.4, 135.7, 134.1, 133.7, 132.5, 131.9, 130.1, 129.6, 129.4, 129.3, 129.1, 127.9, 126.4, 122.8, 110.2, 62.8, 21.1; HRMS (ESI) *m*/*e* calcd. for C_21_H_16_ClNOS (M + H)^+^ 366.0713, found 366.0711.

5-Bromo-3-(*p*-tolyl)-3-(*p*-tolylthio)indolin-2-one (**5ba**). After purification by silica gel column chromatography (PE:EA = 3:1), compound **5ba** was isolated as a white solid (51 mg, 48%); m.p. = 213–215 °C; *R*_f_ (PE:EA = 3:1) = 0.32; ^1^H-NMR (400 MHz, CDCl_3_): δ 7.99 (s, 1H), 7.54 (d, *J* = 8.4 Hz, 2H), 7.43 (d, *J* = 2.0 Hz, 1H), 7.28 (dd, *J* = 8.4 Hz, 2.0 Hz, 1H), 7.19 (d, *J* = 8.4 Hz, 2H), 7.09 (d, *J* = 8.0 Hz, 2H), 6.91 (d, *J* = 7.9 Hz, 2H), 6.55 (d, *J* = 8.3 Hz, 1H), 2.35 (s, 3H), 2.23 (s, 3H); ^13^C-NMR (100 MHz, *d*_6_-DMSO): δ 175.2, 140.5, 139.7, 137.8, 135.7, 133.0, 132.5, 131.7, 129.4, 129.3, 128.5, 127.5, 126.2, 113.4, 111.8, 62.2, 20.8, 20.7; HRMS (ESI) *m*/*e* calcd. for C_22_H_18_BrNOS (M + H)^+^ 424.0365, found 424.0363.

3-(3-Methoxyphenyl)-3-(p-tolylthio)indolin-2-one (**5ca**). After purification by silica gel column chromatography (PE:EA = 5:1), compound **5ca** was isolated as a white solid (35 mg, 39%); m.p. = 175–176 °C; *R*_f_ (PE:EA = 3:1) = 0.33; ^1^H-NMR (400 MHz, CDCl_3_): δ 7.93 (s, 1H), 7.40 (d, *J* = 7.3 Hz, 1H), 7.31–7.11 (m, 4H), 7.11–7.07 (m, 3H), 6.86 (d, *J* = 7.6 Hz, 3H), 6.66 (d, *J* = 7.6 Hz, 1H), 3.81 (s, 3H), 2.21 (s, 3H); ^13^C-NMR (100 MHz, CDCl_3_): δ 177.1, 159.7, 140.1, 139.8, 137.7, 136.2, 130.6, 129.5, 129.2, 128.7, 126.4, 122.6, 120.4, 114.1, 113.7, 109.9, 62.8, 55.3, 55.3, 21.2; HRMS (ESI) *m*/*e* calcd. for C_22_H_19_NO_2_S (M + H)^+^ 362.1209, found 362.1208.

## 5. Conclusions

We have developed a new synthesis of oxindole thioethers by triphenylphosphine-mediated deoxygenation-thiolation of oxindoles with sulfonyl chlorides as sulfenylation reagents. The above reaction was facilitated by iodide anions, possibly due to the in situ conversion of sulfenyl chlorides to the more reactive sulfenyl iodides. Sulfenylation of 3-aryloxindoles required the presence of a base. The use of a transition-metal-free protocol, readily available reagents, and mild reaction conditions allow this protocol more practical to prepare 3-sulfanyloxindoles than traditional methods. This study demonstrated the potential of sulfonyl chlorides as novel, readily accessible, and environmentally friendly sulfenylation reagents for direct thiolation of electron-rich heterocycles.

## Figures and Tables

**Table 1:**
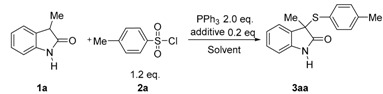
Optimisation of 3-methylindolin-2-one (**1a**) thiolation by 4-methylbenzenesulfonyl chloride (**2a**) in the presence of PPh_3_. *^a^*

Entry	Additive/eq.	Temperature (°C)	Solvent/Volume (mL)	Yield (%) ^b^
1	-	80	1,4-dioxane/1.0	56
2	KI/0.2	80	1,4-dioxane/1.0	56
3	NH_4_I/0.2	80	1,4-dioxane/1.0	73
4	*n*-Bu_4_NI/0.2	80	1,4-dioxane/1.0	82
5	*n*-Bu_4_NI/0.2	80	DCE/1.0	75
6	*n*-Bu_4_NI/0.2	80	toluene/1.0	66
7	*n*-Bu_4_NI/0.2	80	CH_3_CN/1.0	46
8	*n*-Bu_4_NI/0.2	80	DMF/1.0	45
9	*n*-Bu_4_NI/0.2	70	1,4-dioxane/1.0	79
10	*n*-Bu_4_NI/0.2	90	1,4-dioxane/1.0	28
11	*n*-Bu_4_NI/0.2	80	1,4-dioxane/1.5	68
12	*n*-Bu_4_NI/0.2	80	1,4-dioxane/0.5	86
13	*n*-Bu_4_NI/0.2	80	1,4-dioxane/0.3	74
14	*n*-Bu_4_NI/0.2	80	1,4-dioxane/0.5	86 *^c^*

^a^ Reaction conditions: **1a** (0.5 mmol), **2a** (0.6 mmol), PPh3 (1.0 mmol), and additive (0–0.1 mmol)) in an appropriate solvent (0.3–1.5 mL) for 12 h at the indicated temperature. ^b^ Yield of product isolated after silica gel chromatography. ^c^ 2 (0.75 mmol) and PPh3 (1.25 mmol) were used.

**Table 2:**
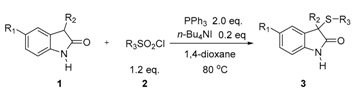
Thiolation of 3-alkyloxindoles with sulfonyl chlorides in the presence of PPh_3_. ^a^

Entry	Oxindole	R_1_	R_2_	Sulfonyl Chloride	R_3_	Product	Yield (%)
1	**1a**	H	Me	**2b**	*p*-MeOC_6_H_4_	**3ab**	90
2	**1a**	H	Me	**2c**	*m*-MeC_6_H_4_	**3ac**	62
3	**1a**	H	Me	**2d**	3,5-Cl_2_C_6_H_3_	**3ad**	68
4	**1a**	H	Me	**2e**	*p*-BrC_6_H_4_	**3ae**	82
5	**1a**	H	Me	**2f**	cyclopropyl	**3af**	44
6	**1a**	H	Me	**2g**	*n*-Butyl	**3ag**	56
7	**1b**	Br	Me	**2a**	*p*-MeC_6_H_4_	**3ba**	90
8	**1c**	H	Et	**2a**	*p*-MeC_6_H_4_	**3ca**	79
9	**1d**	H	Pr	**2a**	*p*-MeC_6_H_4_	**3da**	81
10	**1e**	H	*i*-Pr	**2a**	*p*-MeC_6_H_4_	**3ea**	63 ^b^
11	**1f**	H	*i*-Bu	**2a**	*p*-MeC_6_H_4_	**3fa**	78 ^b^
12	**1g**	H	cyclohexyl	**2a**	*p*-MeC_6_H_4_	**3ga**	67 ^b^
13	**1g**	H	cyclohexyl	**2b**	*p*-MeOC_6_H_4_	**3gb**	65 ^b^
14	**1h**	H	*p*-NCC_6_H_4_CH_2_	**2a**	*p*-MeC_6_H_4_	**3ha**	60 ^b^
15	**1i**	H	*p*-ClC_6_H_4_CH_2_	**2a**	*p*-MeC_6_H_4_	**3ia**	79 ^b^

^a^ Reaction conditions: **1a**–**1d** (0.5 mmol), **2a**–**2g** (0.6 mmol), PPh3 (1.0 mmol), *n*-Bu4NI (0.1 mmol), 1,4-dioxane (0.5 mL), 80 °C, 12 h. ^b^ **1e**–**1i** (0.25 mmol), **2a**–**2g** (0.3 mmol), PPh3 (1.5 mmol), *n*-Bu4NI (0.05 mmol), 1,4-dioxane (0.25 mL), 80 °C, 6-30 h.

**Table 3:**
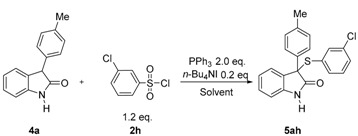
Optimisation of 3-(*p*-tolyl)indolin-2-one (**4a**) thiolation by 3-chlorobenzenesulfonyl chloride (**2h**) in the presence of PPh_3_. ^a^

Entry	Base/eq.	Temperature (°C)	Reaction Time (h)	Solvent/Volume (mL)	Yield (%) *^b^*
1	-	80	15	1,4-dioxane/0.5	0
2	-	60	15	1,4-dioxane/0.5	44
3	-	60	14	1,4-dioxane/1.0	48
4	-	60	20	1,4-dioxane/1.5	39
5	K_2_CO_3_/0.5	60	15	1,4-dioxane/1.0	54
6	K_2_CO_3_/0.5	50	34	1,4-dioxane/1.0	45
7	K_2_CO_3_/0.5	40	38	1,4-dioxane/1.0	71
8	K_2_CO_3_/0.5	25	114	1,4-dioxane/1.0	68
9	Na_2_CO_3_/0.5	40	34	1,4-dioxane/1.0	70
10	Cs_2_CO_3_/0.5	40	39	1,4-dioxane/1.0	26
11	K_2_CO_3_/1.0	40	11	1,4-dioxane/1.0	70
12	K_2_CO_3_/0.5	40	21	1,4-dioxane/1.0	66 *^c^*
13	K_2_CO_3_/0.5	40	51	1,4-dioxane/1.0	27 *^c^*
14	K_2_CO_3_/0.5	40	32	1,4-dioxane/1.0	44 *^d^*

^a^ Reaction conditions: **4a** (0.25 mmol), 2 h (0.3 mmol), PPh3 (0.5 mmol), and *n*-Bu4NI (0.05 mmol) in 1,4-dioxane (0.5–1.5 mL) for indicated time and at specified temperature. ^b^ Yield of product isolated after silica gel chromatography. ^c^ *n*-Bu4NI (0.125 mmol) was used. d2h (0.375 mmol) and PPh3 (0.75 mmol) were used.

## Supplementary Materials

The following are available online, ^1^H-NMR and ^13^C-NMR of compound **3aa**–**3gb** and **5aa**–**5ca.**
